# *Peromyscus* as a Mammalian Epigenetic Model

**DOI:** 10.1155/2012/179159

**Published:** 2012-03-07

**Authors:** Kimberly R. Shorter, Janet P. Crossland, Denessia Webb, Gabor Szalai, Michael R. Felder, Paul B. Vrana

**Affiliations:** Peromyscus Genetic Stock Center and Department of Biological Sciences, University of South Carolina, Columbia, SC 29208, USA

## Abstract

Deer mice (*Peromyscus*) offer an opportunity for studying the effects of natural genetic/epigenetic variation with several advantages over other mammalian models. These advantages include the ability to study natural genetic variation and behaviors not present in other models. Moreover, their life histories in diverse habitats are well studied. *Peromyscus* resources include genome sequencing in progress, a nascent genetic map, and >90,000 ESTs. Here we review epigenetic studies and relevant areas of research involving *Peromyscus* models. These include differences in epigenetic control between species and substance effects on behavior. We also present new data on the epigenetic effects of diet on coat-color using a *Peromyscus* model of agouti overexpression. We suggest that in terms of tying natural genetic variants with environmental effects in producing specific epigenetic effects, *Peromyscus* models have a great potential.

## 1. Introduction

### 1.1. Importance of Epigenetics

Understanding epigenetic effects and their associated gene-environment causes is important in that they are thought to play a large role in human disease susceptibility and etiology. Epigenetic effects are also important in agriculture, evolution, and likely in understanding ecological interactions. Gene-environment interactions are central to the concept of epigenetics, which may be defined as heritable phenotypic changes not mediated by changes in DNA sequence. Research within the last decade has revealed that many classes of genes are subject to epigenetic regulation. Such regulation likely explains much of the lineage/tissue-specific gene expression observed in mammals [1]. For example, several stem cell regulatory loci are regulated in this fashion [2, 3]. Moreover, epigenetic responses to environment, including brief exposures, appear to regulate gene expression involved in many biological processes [4–7].

These environmental response mechanisms inducing epigenetic change are largely unknown. Environmental sensitivity is illustrated by the epigenetic abnormalities seen in cultured mammalian embryos [8–10] and influences of maternal diet and behavior on offspring epigenetic marks such as DNA methylation and histone modifications [11–13]. Therefore, epigenetic effects might be predicted to vary across organisms with diverse life histories and reproductive strategies.

### 1.2. Caveats of Mammalian Systems

Surprisingly, there is no widely used mammalian system for studying epigenetic effects in wild-type genomes. Model systems such as rats, dogs, cows, and sheep do not represent natural populations and have been altered by domestication and other human selection [14]. The most widely used biomedical mammalian model systems are the common inbred strains of laboratory mouse (*Mus*). The common inbred strain genomes differ from wild type in two respects in addition to conscious human selection. First, the complete homozygosity of these strains is not natural. The full scope of changes induced or selected for by inbreeding is not yet known; one that seems highly likely is the presence of highly elongated telomeres in these strains [15] and attenuated behaviors [16].

The final (and perhaps least appreciated) difference of common inbred strain genomes from wild type are the combinations of alleles [17–19] and corresponding patterns of variation. That is, the genome-wide combination of alleles (whether homo- or heterozygous) found in these strains does not exist in nature. Moreover, recent studies show that the genetic diversity found in the inbred strains is limited [20]. That is, the genetic architecture of model systems does not resemble humans [21]. An obvious solution that has been proposed is to incorporate more wild-derived/non traditional systems [16, 20].

### 1.3. Introduction to Peromyscus and the PGSC

**Figure 1:**
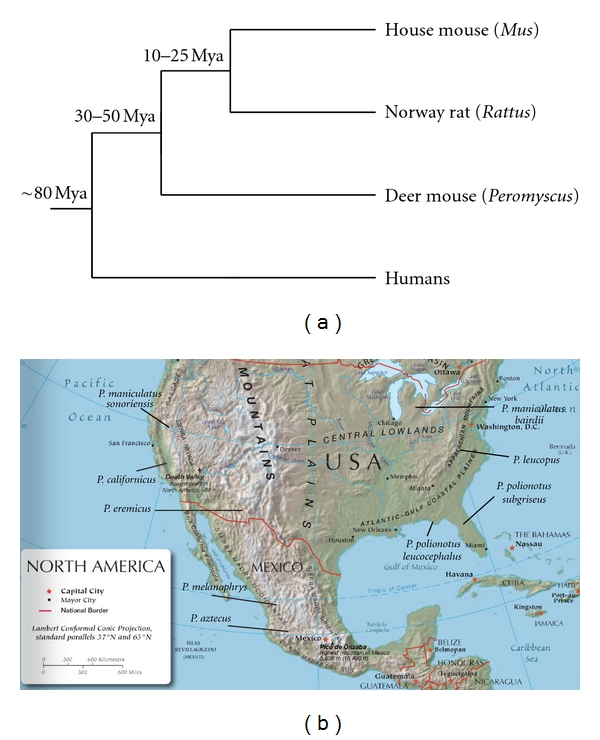
(a) Phylogenetic placement of *Peromyscus* and approximate divergence times from laboratory mice, rats, and humans. (b) Map showing locales where PGSC stocks' founders were caught.

The rodent genus *Peromyscus*, colloquially termed deer- or field-mice, is the largest and most wide-spread group of indigenous North American mammals [22]; the group's 55+ species are found in every terrestrial ecosystem. Despite superficial resemblances, these animals represent a relatively old divergence (30 to 50 MYA) from both *Mus* and rats (*Rattus*) within the muroid rodents [23] (Figure 1(a)). Most of these species are easy to capture and breed well in captivity, facilitating study of natural variants.

The major stocks maintained by the *Peromyscus* Genetic Stock Center (PGSC; http://stkctr.biol.sc.edu/) are wild-derived. That is, a number of founder animals were caught at a specific locale over a short time period, and their random-bred descendants are considered a single stock. Among these are three of the few species of mammals which have shown to be monogamous and to exhibit pair bonding (*P. californicus*, *P. polionotus*, and *P. eremicus*).  Figure 1(b) depicts the origins of these major stocks. The additional natural variants and mutants housed by the PGSC have typically been bred onto the *P. maniculatus bairdii* (BW; http://stkctr.biol.sc.edu/wild-stock/p_manicu_bw.html) stock genetic background.

**Figure 2:**
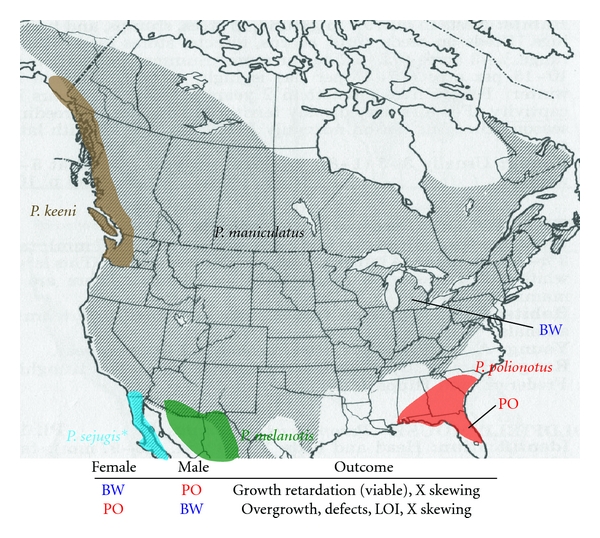
*Peromyscus maniculatus* species complex, captive stock origins, and cross results. Ranges are indicated by color, except *P. maniculatus*, which is shaded. *P. sejugis range includes adjacent *P. maniculatus* populations which exhibit greater affinities to this species [31, 32]. Ranges of *P. keeni*, *P. maniculatus*, *P. melanotis,* and *P. sejugis* extend beyond map. LOI: Loss of (genomic) imprinting; X skewing: skewing of X chromosome during inactivation in somatic tissues. Studies from the 1930s–1950s period suggest asymmetries in crosses between other populations/species (i.e., besides PO and BW).

The *Peromyscus maniculatus* species complex is particularly wide-spread and variable across North America (Figure 2). Viable and fertile interspecific hybrids are possible between many populations and species within this group (e.g., *P. maniculatus* females × *P. polionotus* males). Due to these factors, the majority of resource development has occurred within this group. These resources include a recently completed genetic map of *P. maniculatus* (BW stock)/*P. polionotus* (PO stock; http://stkctr.biol.sc.edu/wild-stock/p_polion_po.html), ~90,000 ESTs to date (additional transcriptome data of other organs will follow), and completed sequencing of both the BW and PO genomes. Assembly of these two genomes is in progress. Genome sequencing of two additional species, *P. leucopus* (also quite widespread in North America, and exceptionally long-lived [22, 24–26]) and *P. californicus* (arguably the best known mammalian monogamy model [27–29]) will follow.

Further, major advances have been made in reproductive manipulation of *P. maniculatus* [30]. We have greatly increased the number of oocytes/embryos recovered after induced ovulation. Second, we have also optimized conditions for culturing embryos. These advances (1) allow for easier study of early developmental stages, (2) allow a greater chance for success in cryopreservation, and (3) allow embryo manipulation (e.g., transgenics, chimera production).

Here we review epigenetic studies and relevant areas of research involving *Peromyscus* models as well as presenting new data on the epigenetic effects of diet on coat-color using a *Peromyscus* model of agouti overexpression.

## 2. Incompatibility between *P. polionotus* and *P. maniculatus* Epigenetic Regulation

### 2.1. Epigenetics in Mammalian Reproductive Isolation

An emerging theme in mammalian development is the involvement of epigenetic control of key regulatory loci [1, 2, 33–36]. The epigenetic modifications at these loci are of the same type as those observed at imprinted loci, retroelements (i.e., to prevent their transcription), the inactive X-chromosome, and in heterochromatin [37–39]. Therefore, changes in epigenetic regulation could both alter development and contribute to reproductive isolation.

Reproductive isolation is thought to be driven by sets of interacting loci in which derived allele combinations are deleterious [40]. One approach to studying such variants is to utilize interspecific hybrids, which exhibit dysgenic or maladaptive phenotypes [41]. A number of studies have employed such hybrids to map and identify the causative loci [42–45]. However, the few studies in mammals largely involve hybrid sterility [46] and thus offer little information on genes involved in developmental isolating mechanisms. Despite the lack of mapping studies, epigenetic mechanisms have been implicated in mammalian reproductive isolation in several cases, including (a) Gibbon (*Nomascus*) karyotypic evolution [47], (b) hybrid sterility between the house mouse species *Mus musculus*—*M. domesticus* [48], (c) retroelement activation in both Wallaby (*Macropus*) [49], and (d) *Mus musculus*—*M. caroli* hybrids [50].

The *Peromyscus maniculatus* species complex of North America offers great potential for such genetic studies [14]. Among the many variable characteristics in this group are the heterochromatic state of some genomic regions [51, 52]. This heterochromatin variation itself indicates epigenetic variation. Interspecific crosses within this group exhibit great variation in offspring viability. The best characterized of these are the asymmetries in crosses between *P. maniculatus* (particularly *P.m. bairdii*, the prairie deer mouse; BW stock) and *P. polionotus *(PO stock) [53–56], whose range is significantly more limited (Figure 2). One potential explanation of such asymmetries involves genes subject to the epigenetic phenomenon of genomic imprinting, which is the differential expression of the two parental alleles of a given locus.

### 2.2. Genomic Imprinting

Demonstration of the epigenetic nonequivalence of mammalian maternal versus paternal genomes [57–59] led to the discovery of imprinted loci. Imprinted genes exhibit biased allelic expression dependent on parental origin. That is, some loci are silenced during oogenesis and others during spermatogenesis. Differential allelic DNA methylation of cytosine residues is thought to be the primary epigenetic mark responsible for genomic imprinting [60–62]. These discrete differentially methylated regions (DMRs) arise in gametogenesis, where the responsible epigenetic marks must be reset [63–65]. DNA methylation at these DMRs survives the global genomic demethylation during embryogenesis [66–68] and may have long-range effects on gene expression [69].

### 2.3. Loss of Imprinting in Peromyscus Hybrids

*P. maniculatus* females × *P. polionotus* males (♀bw × ♂po, so denoted to indicate the growth retardation outcome of the cross) produce growth-retarded, but viable and fertile offspring [55, 70, 71]. The ♀bw × ♂po hybrids display few alterations in imprinted gene allelic usage or expression levels [72, 73]. For example, the *Igf2r* gene shows slight reactivation of the normally silent paternal allele in ♀bw × ♂po extraembryonic tissues. The product of this gene negatively regulates the Insulin-like Growth Factor 2 (*Igf2*) protein. The growth-retarded hybrids also exhibit lower levels of the imprinted *Igf2* transcript in embryonic and placental tissues at some time points [73, 74]. However, normal *Igf2* paternal expression is maintained.

In contrast, *P. polionotus* females × *P. maniculatus* males (♀PO × ♂BW) produce overgrown but dysmorphic conceptuses. Most ♀PO × ♂BW offspring are dead by mid-gestation; those surviving to later time points display multiple defects [73]. A portion (~10%) of ♀PO × ♂BW conceptuses consist of only extraembryonic tissues, indicating major shifts in cell-fate. Roughly a third of pregnancies have one or more live embryos at this age. Most of these embryos have visible defects that suggest nonviability (e.g., hemorrhaging) [73]. The rare ♀PO × ♂BW litters that reach parturition typically result in maternal death due to inability to pass the hybrid offspring through the birth canal [75].

**Figure 3:**
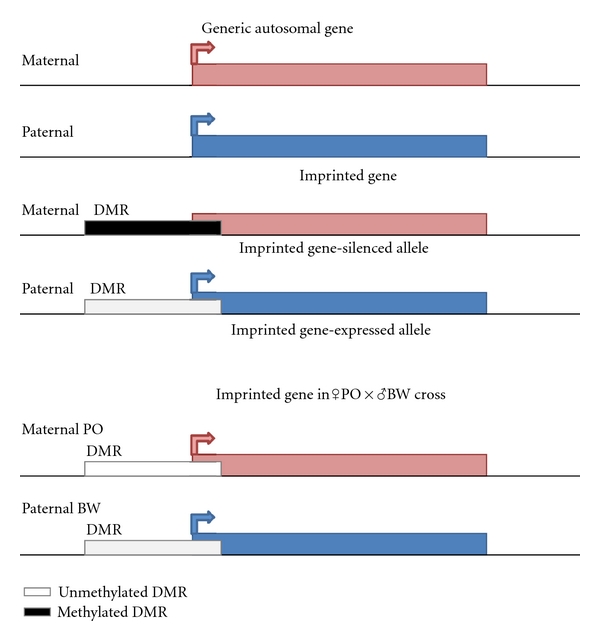
Diagram of effects of hybridization on genomic imprinting. A generic autosomal gene expressed from both parental alleles is shown on top. An imprinted gene expressed from the paternal allele with a methylated DMR on the silenced maternal allele is shown in the middle. The same (normally imprinted) gene losing imprinting and DMR methylation in the ♀PO × ♂BW offspring is shown at the bottom.

Our research has shown that many loci lose imprinted status and associated DMR DNA methylation in the ♀PO × ♂BW hybrids [72, 73, 76, 77] (Figure 3). While the extent of ♀PO × ♂BW DNA methylation loss is not known, restriction digests suggest it is not genome-wide. Excluding a *Peromyscus*-specific prolactin-related placental lactogen, which displays paternal expression [76], we have tested the expression of over twenty known imprinted genes in the hybrids [77]; the majority exhibit hybrid perturbations. In the case of *H19* and *Igf2*, two tightly linked loci are differentially affected. *H19* loses imprinting (and exhibits higher expression levels), while neither *Igf2* allelic expression nor levels have been affected in the ♀PO × ♂BW hybrids examined [72, 73]. Also pure strain PO and BW embryos exhibit significantly different expression levels of some imprinted genes (*Igf2*, *Grb10)* [73].

Two imprinted loci contribute to the ♀PO × ♂BW overgrowth: *Mexl *(maternal effect X-linked) and *Peal* (paternal effect autosomal locus) [78, 79]. The *Mexl*-*Peal* interactions do not account for the loss of genomic imprinting or the developmental defects. Rather, these effects are due to the *Meil* (maternal effect on imprinting locus) locus where the effect is dependent on maternal genotype [80]. Females homozygous for the PO *Meil* allele produce the severe dysgenesis in their offspring when mated to BW males. The imprinted genes perturbed in the ♀PO × ♂BW cross do not match the patterns displayed by targeted mutations of any of the DNA methyltransferase encoding (*Dnmt*) loci [80], though those also produce maternal effects [81–84].

### 2.4. Hybrid X Inactivation

Both hybrid types display skewed X-chromosome inactivation in somatic tissues [78]. That is, the PO allele is preferentially silenced. This difference is mediated by the X-chromosome inactivation center. Surprisingly, imprinted X-inactivation, in which the paternally-inherited X is silenced, is maintained in the extraembryonic tissues of both hybrid types. Note that paternal X inactivation is believed to be the default and ancestral state in mammals [85, 86].

Thus it is clear that epigenetic control of individual loci as well as genome-wide epigenetic control differs between *P. maniculatus* and *P. polionotus*. We suggest that this may be the case between other species within the *P. maniculatus* species complex [14].

### 2.5. Use of Peromyscus in Other Genomic Imprinting/X Chromosome Studies

The frequent polymorphisms between the two species has facilitated the discovery of novel imprinted loci. A screen in the lab of SM Tilghman used a differential display approach on PO, BW, and reciprocal hybrid placental tissues which led to the discovery of imprinting of *Dlk1*, *Gatm,* and a *Peromyscus*-specific placental lactogen encoding gene. [76, 87, 88]. However, many of the putative newly discovered imprinted loci were never vetted.

The phylogenetic placement of *Peromyscus* (more divergent from lab rats and mice, Figure 1(a)) renders them useful for evolutionary studies. Several studies have shown absence of genomic imprinting at specific loci (*Rasgrf1*, *Sfmbt2*) in *Peromyscus* along with absence of putative regulatory elements, thereby strengthening the mechanistic hypotheses [89, 90].

A recent study utilized animals of the PGSC *P. melanophrys* (XZ) stock to investigate reports of anomalous sex chromosomes in this species [91]. Using *P. maniculatus* chromosome paints, they identified a region common to both the X and Y chromosomes, which has translocated to an autosome. This region has some characteristics of the inactive X chromosome (e.g., late-replication) but lacks others such as trimethyl-H3K27 modification [92].

## 3. Effects of a High-Methyl Donor Diet on the *Peromyscus* Wide-Band Agouti Phenotype

### 3.1. The Agouti A^vy^ Allele and Epigenetics

The best studied example of dietary effects on mammalian epigenetics concerns the viable yellow allele of the agouti locus (A^vy^) in laboratory mice [11, 93]. The A^vy^ allele displays variable misexpression due to the insertion of an intracisternal A particle (IAP) retroviral-like element 5′ of the agouti promoter. Overexpression of agouti results in obesity and cancer susceptibility as well as a yellow coat-color [94, 95]. The latter phenotype differs from that of wild-type mice, whose individual hairs exhibit bands of yellow and brown (as do those of many mammals).

Maternal diets supplemented with additional methyl-donor pathway components (all taken as human dietary supplements) result in A^vy^ offspring with wild-type coloration and adiposity [11, 93]. This rescue occurs in spite of the fact that these animals are genetically identical to unrescued animals. These effects are due to the selective DNA methylation (and hence silencing) of the IAP element promoter. A maternal diet with a greater amount of supplementation resulted in a greater reduction of the abnormal phenotypes.

A nearly identical phenomenon has been documented with a lab mouse variant of the Axin gene. An IAP insertion into an Axin intron resulted in the fused allele (Axin^Fu^) [96]. The IAP element results in a truncated protein, which interferes with the WT product's role in axial patterning. Thus Axin^Fu^ animals have a variable degree of tail-kinking.

A high methyl-donor maternal diet identical to that used in the A^vy^ studies (which of the two diets is not specified) results in lower incidence and less severe tail-kinking. The rescued Axin^Fu^ offspring also exhibits greater methylation of the IAP retroelement. Further, the tail appears to be more labile than the liver in terms of DNA methylation at this allele. These findings have particular import if such gestational dietary modification promotes methylation at loci other than these unusual IAP alleles.

### 3.2. Effects of Diet on the Peromyscus A^Nb^ Allele

To test the hypothesis that such a diet may not only affect IAP elements, we utilized the same high methyl-donor chow used in the agouti A^vy^ and Axin^Fu^ studies (Table 1). We employed a naturally occurring *Peromyscus* allele, which overexpresses agouti, termed wide-band agouti (A^Nb^; http://stkctr.biol.sc.edu/mutant-stock/wide_band.html) [97–99]. We mated standard BW females to homozygous A^Nb^ males and analyzed the resulting offspring either fed a normal diet (Harlan 8604 Teklad Rodent Diet; http://www.harlan.com/) or the methyl-donor-enriched diet Harlan Teklad TD.07517 Methyl Diet; the latter is the “MS” diet used in prior methyl-donor diet studies [11, 93]. A comparison between this diet and the standard chow is shown in Table 1. Offspring were fed the same diet postweaning, until sacrificed at ~six months of age (when coat color is mature; note that these animals live >4 years). After being euthanized, the animals were skinned, and tufts of hair were analyzed by light microscopy.

**Figure 4:**
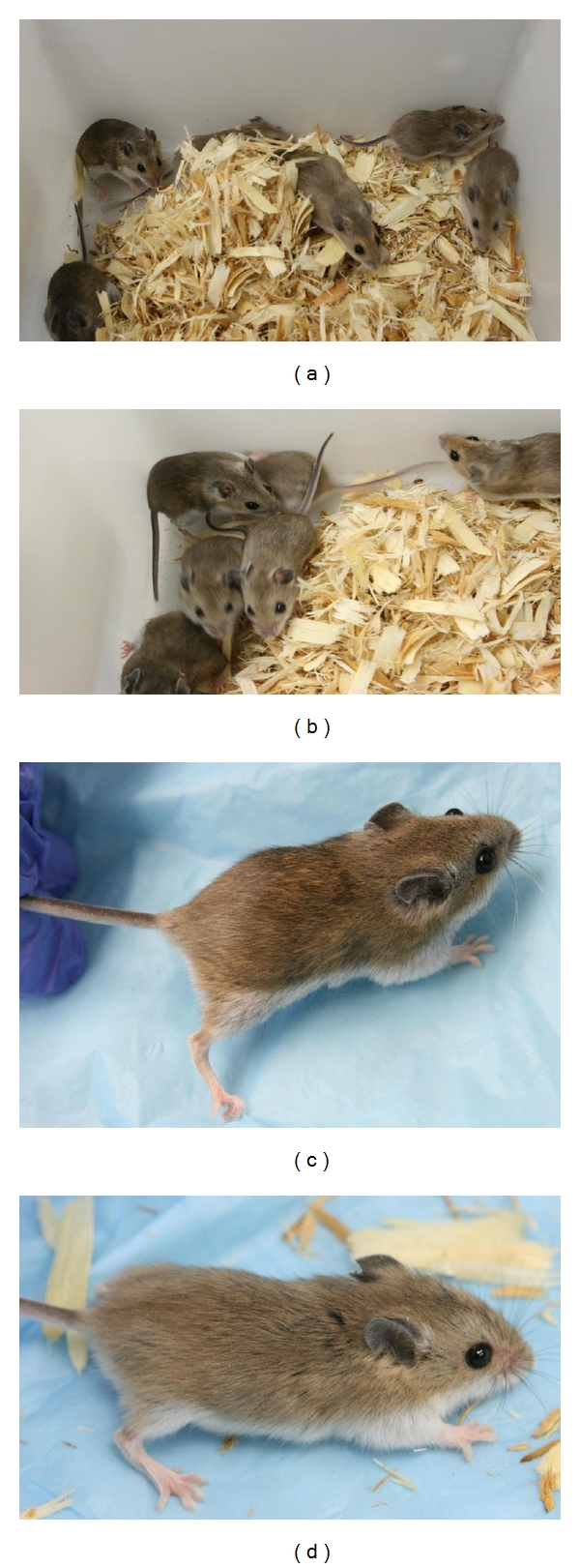
(a, b) Heterozygous wide-band Agouti (A^Nb^) litters exposed to methyl-donor diet; note variation. (c) Individual from litter shown in (a, b). (d) Age-matched heterozygous A^Nb^ animal exposed only to standard rodent chow. All animals shown within 4 days post weaning (24–28 days postnatal). All animals used in laboratory studies presented were bred or derived from stocks kept at the *Peromyscus* Genetic Stock Center. Most species (including all those discussed below) may be kept in standard mouse cages. *Peromyscus* are kept on a 16 : 8 light/dark cycle (rather than a 12 : 12 cycle) to facilitate breeding. All experiments presented were approved by the University of South Carolina Institutional Animal Care and Use Committee (IACUC).

Whereas A^Nb^ heterozygous animals are uniformly light in coloration (Figure 4(d)), we observed large variability in the animals whose mothers were fed the methyl-donor diet as soon as weaning (Figures 4(a) and 4(c)). Thus the maternal diet alone can affect the status of a non-IAP-regulated agouti locus.

**Figure 5:**
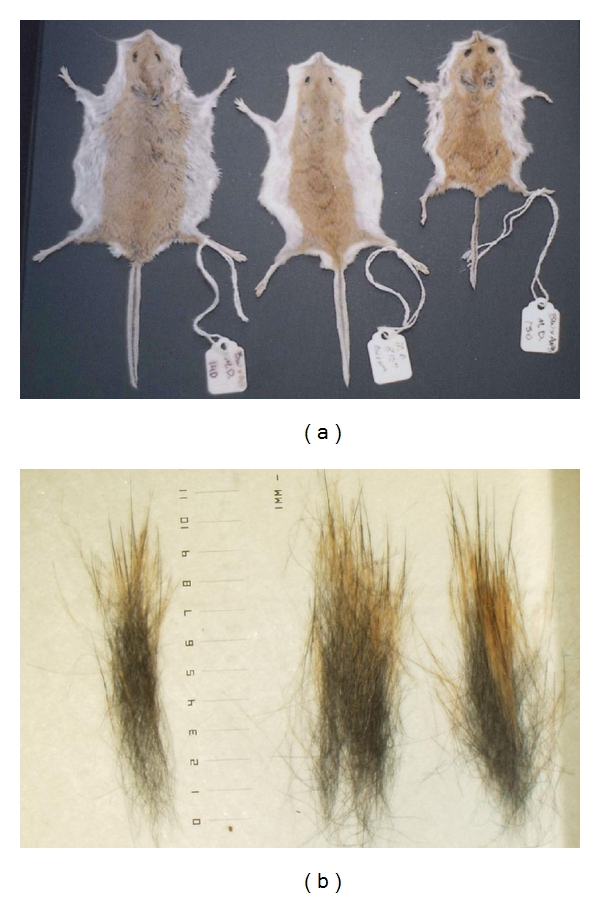
Pelts and hair clumps from selected heterozygous wide-band Agouti (A^Nb^) raised on the methyl-donor diet. (a) Pelts from animals exhibiting differential coat-colors. (b) Microscopy of dorsal hair clumps from same animals (and same order) as in (a). Note the longer yellow band in the rightmost sample.

Analysis of the coats of other animals at six months of age (maintained on the diet) confirmed this variation in animals exposed to the methyl-donor-enriched diet. Some animals had a yellow hair band of only 2-3 mm, whereas this band extended to 5-6 mm in other animals. This length corresponds to the overall appearance of the coat (i.e., the longer the band, the lighter the coat, Figure 5). Future studies will examine the DNA methylation status of the agouti gene and other loci in these animals as well as potential behavioral effects.

## 4. Toxicology and Epigenetics

### 4.1. Peromyscus as a Toxicology Model

Due to their ubiquity, *Peromyscus* are found in most North American contaminated (e.g., due to mining or manufacturing) sites, even where other animals are absent [100–102]. Comparison of PGSC animals exposed to these compounds is a fruitful way to study the physiological consequences of xenobiotic exposure. One unexplored research avenue is whether animals at sites contaminated with heavy metals exhibit epigenetic changes, as cadmium and nickel (among others) have been shown to induce such change [103–106].

Stock Center animals have been employed for studies involving PCBs [107–112], 4,4′-DDE [113], Aroclor 1254 [114, 115], 2,4,6-trinitrotoluene [116], ammonium perchlorate [117], and RDX [118–120]. One of the PGSC stocks has a natural deletion of the alcohol dehydrogenase (ADH) gene [121], which has proven useful for delimiting the relative roles of ADH and microsomal oxidases in ethanol metabolism [122] and the metabolic basis of ethanol-induced hepatic and pancreatic injury [123]. Ethanol and its metabolites have also been associated with changes in epigenetic marks [124, 125].

### 4.2. BPA Peromyscus Studies

Bisphenol A (BPA) is a chemical used in the production of poly-carbonate plastic and epoxy resins. BPA is commonly used in products including food and beverage containers, baby bottles and dental composites; it is present in 93% of human urine samples in the United States and is a known endocrine disruptor [126]. Dolinoy and colleagues found that prenatal exposure to BPA through maternal dietary supplementation (50 mg/kg) produced significantly decreased methylation of nine sites of the A^vy^ locus, as well as at the CDK5 activator-binding protein locus [127]. Coat color distribution was shifted towards the yellow coat color phenotype.

A 2011 study demonstrated behavioral disruptions in BW animals by bisphenol A (BPA). BPA altered certain behaviors in male offspring of mothers administered BPA during pregnancy. Specifically, these males had decreased spatial navigational ability and exploratory behavior, traits necessary for finding a mate. Females also preferred non-BPA exposed males, despite the lack of detectable physical effects on the BPA-exposed males [128]. This study, therefore, has broad implications for the effects of these compounds on mammals.

## 5. Additional Areas of *Peromyscus* Epigenetic Study

There are several additional areas of research where *Peromyscus* models appear to have potential.

As noted, *P. leucopus *is a model for ageing, as they live >8 years, ~3-4 times longer than other rodents of comparable size. That longevity is associated with increased vascular resistance to high glucose-induced oxidative stress and inflammatory gene expression [25] and a relatively slower rate of loss of DNA methylation [26].

*P. maniculatus* has a propensity to perform repetitive movements, for example, jumping, whirling, and back flipping [129]. Such behaviors are not only representative of a number of human disorders [130] but also an issue in captive animal welfare. Thus the PGSC BW stock of *P. maniculatus* has become recognized as a model for stereotypy [131]. Attenuation of stereotypy was seen after environmental enrichment [132], suggesting a potential epigenetic effect.

Further, BW populations can be grouped into high and low stereotypic behavior groups, with high and low doses of fluoxetine reducing the phenotype in both groups [133]. The two stereotypy levels found in the BW population make them a model for basic research on brain function during repetitive motion and also provide a model for gene-environment epimutation analysis.

## 6. Conclusions

The interplay between environment and genotype that results in specific epigenetic changes appears complex. *Peromyscus* offers the opportunity to study natural genetic variants in both laboratory and natural settings and the ability to examine mechanistic and evolutionary aspects of changes in epigenetic control. We suggest that in terms of natural genetic variation and associated epigenetic effects, *Peromyscus* models have a potential not yet realized with any mammalian system. We encourage anyone interested in the possibility of using these animals in their research program to contact the PGSC (http://stkctr.biol.sc.edu/).

## Figures and Tables

**Table 1:** 

	8604	7517
Betaine	0	5
Choline	2.53	7.97
Folic acid	0.0027	0.0043
Vitamin B12	0.051	0.53

Comparison of standard (8604) and Methyl-Donor (7517) diet components (g/Kg of chow).
